# Insular cortex stimulation alleviates neuropathic pain via ERK phosphorylation in neurons

**DOI:** 10.1111/cns.14126

**Published:** 2023-02-20

**Authors:** Kyeongmin Kim, Guanghai Nan, Leejeong Kim, Minjee Kwon, Kyung Hee Lee, Myeounghoon Cha, Bae Hwan Lee

**Affiliations:** ^1^ Department of Physiology Yonsei University College of Medicine Seoul Korea; ^2^ Department of Medical Science, Brain Korea 21 Project Yonsei University College of Medicine Seoul Korea; ^3^ Department of Nursing Kyungil University Gyeongsan Korea; ^4^ Department of Dental Hygiene, Division of Health Science Dongseo University Busan Korea

**Keywords:** extracellular signal‐regulated kinase, insular cortex, insular cortex stimulation, neuron, neuropathic pain

## Abstract

**Aims:**

The clinical use of brain stimulation is attractive for patients who have side effects or tolerance. However, studies on insular cortex (IC) stimulation are lacking in neuropathic pain. The present study aimed to investigate the effects of IC stimulation (ICS) on neuropathic pain and to determine how ICS modulates pain.

**Methods:**

Changes in pain behaviors were observed following ICS with various parameters in neuropathic rats. Western blotting was performed to assess molecular changes in the expression levels of phosphorylated extracellular signal‐regulated kinase (pERK), neurons, astrocytes, and microglia between experimental groups. Immunohistochemistry was performed to investigate the colocalization of pERK with different cell types.

**Results:**

The most effective pain‐relieving effect was induced at 50 Hz–120 μA in single trial of ICS and it maintained 4 days longer after the termination of repetitive ICS. The expression levels of pERK, astrocytes, and microglia were increased in neuropathic rats. However, after ICS, the expression levels of pERK were decreased, and colocalization of pERK and neurons was reduced in layers 2–3 of the IC.

**Conclusion:**

These results indicated that ICS attenuated neuropathic pain by the regulation of pERK in neurons located in layers 2–3 of the IC. This preclinical study may enhance the potential use of ICS and identify the therapeutic mechanisms of ICS in neuropathic pain.

## Graphical abstract

The most effective alleviation effect in neuropathic pain is induced by the insular cortex stimulation (ICS) at 50 Hz–120 μA and maintained. In the IC, the expression of phosphorylated extracellular signal‐regulated kinase was colocalized with neurons in neuropathic pain and reduced after ICS.

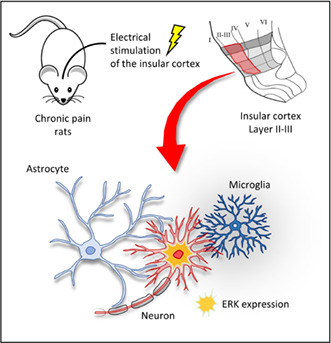

## 1 INTRODUCTION

Damaged or dysfunctional nerves due to various causes such as infection or injury can cause the neuropathic pain (NP).^1^, ^2^ Representative symptoms of NP include allodynia, hyperalgesia, and spontaneous pain, which adversely affect 10%–20% of the whole population and lead to staggering health care costs.^3^ Although many treatments have been developed to treat NP, patients still experience the side effects or tolerance due to long‐term use.^3^, ^4^ For these patients, neurostimulation techniques such as transcranial magnetic stimulation and deep brain stimulation are required as progressive surgical therapies for the management of malignant pain.^5^, ^6^ However, only a few regions of the brain, for instance, the motor cortex, anterior cingulate cortex, and thalamus, have been investigated for the clinical trials of brain stimulation in NP,^7^, ^8^, ^9^ and the parameters of brain stimulation are used in various range.^8^, ^9^, ^10^ Also, the basic and therapeutic mechanisms of brain stimulation remain unknown. Although a few studies^11^, ^12^, ^13^ have examined the attenuation effects and mechanisms of the insular cortex (IC) stimulation (ICS), no studies have been revealed the optimal parameters of ICS that induce the most improved alleviation effect in NP.

The IC is activated by noxious stimulation and contributes to unpleasant feelings of pain.^14^, ^15^ In NP state, the activation and the responsiveness of the IC are excessively elevated compared with those in the normal state, and modulated by mammalian target of rapamycin complex or fatty acid amide hydrolase to ameliorate NP.^16^, ^17^, ^18^, ^19^ Moreover, previous studies have reported that IC contributes to alteration of opioid receptor or γ‐aminobutyric acid neurotransmission for pain modulation.^20^, ^21^ These studies suggest that the IC is involved in the modulation of physiological or pathological pain.

Neuropathic pain induces dynamic changes at molecular or synaptic levels and alters intracellular signal transduction or numerous cascade molecules in the nervous system.^2^, ^22^ Among them, extracellular signal‐regulated kinase (ERK) is one of the kinases activated by signal transduction and is involved in the maintenance of central sensitization.^23^ Many studies have reported that ERK is selectively phosphorylated and expressed depending on the intensity of noxious stimuli^24^, ^25^ and direct inhibition of phosphorylated ERK (pERK) expression alleviates NP.^24^, ^26^ Owing to these characteristics, pERK could be used as a marker of nociceptive activity.^23^, ^25^ In addition, pERK is expressed in neurons, astrocytes, and microglia depending on the pain development, target regions, and types of chronic pain models.^26^, ^27^, ^28^ The interaction of pERK with neurons and glial cells in NP leads to enhanced pain states by strengthening the synaptic plasticity.^29^ However, no studies have revealed the distribution of pERK in neurons or glial cells after ICS in relation to NP.

Therefore, we aimed to investigate the most effective parameters and alleviation effect of ICS for controlling NP. This study also aimed to elucidate which cell types interact with ERK phosphorylation in the IC under NP states or after ICS.

## 2 MATERIALS AND METHODS

### 2.1 Experimental animals

All animal experimental procedures adhered to the National Institutes of Health guidelines, and the experimental protocols were approved by the Institutional Animal Care and Use Committee of the Yonsei University Health System (permit no. 2019‐0225). Male Sprague–Dawley rats (weight, 240–260 g; Harlan, KOATEC, Pyeongtaek, Korea; *n* = 280) were used in all experiments. Three rats were housed per cage under a 12‐h dark/light cycle, with food and water available ad libitum. The groups were divided into sham surgery with sham stimulation (sham injury group), neuropathic surgery with sham stimulation (NP group), and neuropathic surgery with ICS (ICS group).

### 2.2 Electrode implantation, neuropathic surgery, and behavioral test

Under sodium pentobarbital anesthesia (50 mg/kg, intraperitoneal injection [i.p.]), rats were positioned on a stereotaxic frame. Atropine (5 mg/kg, i.p.) was administered to rats to prevent mucus secretion. The stimulation electrode (MS308, P1 Technologies, Roanoke, VA, USA) was unilaterally implanted into the rostral IC (1.0 mm anterior to bregma, 5.0 mm lateral from the midline, 7.7 mm below the surface of the skull) of the contralateral side of the nerve‐injured hind paw.

Rats had convalescence for 3 days after electrode implantation. Neuropathic surgery was performed as previously described.^30^ The induction chamber was filled with 5% isoflurane for anesthesia. After incision of the skin and muscles, the tibial and sural nerves were tightly ligated with 5‐0 black silk and sectioned while the common peroneal nerve was kept intact. The rats in the sham injury group underwent identical surgery without nerve injury.

Each rat was positioned in an acrylic cage for 15 min to get habituated to the environment. Behavioral test was performed using an electronic von Frey filament (no. 38450; UGO Basile, Varese, Italy). The filament was applied to the injured side of the hind paw, and force values were measured until the animal exhibited the hind paw withdrawal. The assessment of force values was repeated seven times at 2‐ to 3‐min intervals and averaged. All behavioral tests were conducted in a double‐blind fashion.

### 2.3 ICS

The electrode was connected to a stimulator (A385, WPI, Sarasota, FL, USA) for single or repetitive ICS. Single ICS was applied for 30 min at once on POD 7. The repetitive ICS was applied for 30 min at once a day from POD 7 to 14 and it was terminated from POD 15 to 20. The dependence of ICS efficacy on the stimulation frequencies (25, 50, and 130 Hz) and intensities (5, 10, 40, and 120 μA) were tested based on previous studies.^8^, ^11^, ^12^, ^13^ The pulse width was fixed at 200 μs. In sham injury and NP groups, the electrode was connected to a stimulator without electrical stimulation.

### 2.4 Western blot

On postoperative days (POD) 14, the targeted IC tissues were collected immediately after repetitive trials of ICS or sham stimulation. The collected tissues were immediately frozen in liquid nitrogen and phosphatase inhibitors (PhosSTOP; Roche, Mannheim, Germay) were added to the lysis buffer (PRO‐PREP; Intron Biotechnology, Pyeongtaek, Korea). The samples were homogenized and centrifuged at 22,176 × *g* for 10 min. Protein samples were denatured, separated, and transferred onto membranes (Merck Millipore, Darmstadt, Germay). The membrane was blocked in a 5% bovine serum albumin solution for 1 h at room temperature (RT). After then, the membrane was incubated with primary antibodies for binding to a particular protein overnight at 4°C and also incubated with a secondary antibody (Table 1) for 2 h at RT. The band intensity was quantified using the LAS system (LAS4000, Fuji Film Inc., Tokyo, Japan).

**TABLE 1:** List of antibodies used.

Purpose	Name of antibody	Manufacturer	Catalog number	Species	Monoclonal/polyclonal	Dilution
Western Blot						
Primary antibody	pERK	Cell Signaling Technology	No. 4376	Rabbit	Monoclonal	1:10,000
	ERK	Cell Signaling Technology	No. 4695	Rabbit	Monoclonal	1:15,000
	NeuN	Abcam	ab104224	Rabbit	Polyclonal	1:5000
	GFAP	Abcam	ab7260	Rabbit	Polyclonal	1:10,000
	CD11b/c	BD PharMingen	No. 550299	Mouse	Monoclonal	1:2000
	GAPDH	ABFrontier	LF‐PA0018	Rabbit	Polyclonal	1:10,000
Secondary antibody	Anti‐rabbit	Cell Signaling Technology	No. 7074	Goat	–	1:5000
	Anti‐mouse	Cell Signaling Technology	No. 7076	Horse	–	1:5000
Immunohistochemistry						
Primary antibody	pERK	Cell Signaling Technology	No. 4376	Rabbit	Monoclonal	1:200
	NeuN	Abcam	ab104224	Mouse	Monoclonal	1:1000
	GFAP	Cell Signaling Technology	No. 3670	Mouse	Monoclonal	1:1000
	CD11b/c	BD PharMingen	No. 550299	Mouse	Monoclonal	1:100
Secondary antibody	Cy3	Jackson ImmunoResearch	716‐166‐151	Mouse	–	1:1000
	Alexa Fluor 488	Jackson ImmunoResearch	711‐545‐152	Rabbit	–	1:1000

### 2.5 Immunohistochemistry and image acquisition

On POD 14, the rats were deeply anesthetized with urethane (1.25 g/kg, i.p.) immediately after repetitive trials of ICS or sham stimulation. The anesthetized rats were transcardially perfused with normal saline (0.9% NaCl) followed by 4% paraformaldehyde in 0.1 M sodium phosphate buffer (PB, pH 7.4). For postfixation, the brains were soaked in 30% saccharose in phosphate‐buffered saline (PBS) and cryosectioned to a thickness of 20 μm using a cryostat (HM525, Thermo Scientific, Waltham, MA, USA). Brain sections were washed with PBS and blocked in 10% normal donkey serum (017‐000‐121; Jackson ImmunoResearch, West Grove, PA, USA) in 0.3% Triton X‐100 for 1 h at RT. Brain sections were incubated overnight at 4°C with primary antibodies (Table 1) and rinsed with PBS containing 0.3% Tween‐20. A mixture of secondary antibodies (Table 1) was incubated for 2 h at RT. All sections were mounted with 4′,6‐diamidino‐2‐phenylindole (DAPI, Vector Laboratories, Burlingame, CA, USA). Images were captured using a laser scanning confocal microscope (LSM 700; Carl Zeiss, Jena, Germany). Images were subjected to maximum intensity projection (MIP) using Zen Black software (Carl Zeiss) to represent the branches of glial cells.

### 2.6 Quantification of cell number in the IC

To quantify the cell number in the IC, 10–12 segments (for 5–6 rats) for each group were used to perform the morphometric analyses. An image at 40× magnification was captured and the number of immune‐positive cells was quantified within a 1024 × 1024 pixel sample of each target region of interest.

### 2.7 Quantification of the morphological properties of astrocytes and microglia

The total length and volume of astrocytes were quantified using the Sholl analysis.^31^ The Simple Neurite Tracer plugin (http://fiji.sc/Simple_Neurite_Tracer) for Sholl analysis was applied to the MIP images as previously described.^31^ Astrocytes with DAPI‐stained nuclei and without truncated processes were considered for reconstruction and were semi‐automatically traced by the software. Volume was measured by filling all the paths of reconstructed astrocytes, and a threshold of 0.05 yielded reproducible results for volume analysis. For the morphological analysis of microglia, FIJI‐ImageJ software was used for quantification, as described previously.^32^ To acquire the total process length of microglia, the AnalyzeSkeleton (2D/3D) plugin (http://imagej.net/AnalyzeSkeleton) was used.^32^

### 2.8 Statistical analysis

Statistical analyses were performed using GraphPad Prism software (GraphPad Software). Data were tested for normality using D'Agostino–Pearson test before analysis. Behavioral data for the measurement of mechanical withdrawal thresholds (MWTs) were analyzed using two‐way analysis of variance (ANOVA) with repeated measures followed by Bonferroni's test for post hoc comparisons. Western blot data were analyzed using one‐way ANOVA followed by Bonferroni's post hoc test for multiple comparisons. Confirmation of bivariate associations in immunohistochemistry was analyzed using the chi‐squared test. Morphometric data were analyzed using one‐way ANOVA followed by Bonferroni's post hoc test. Normality test with the D'Agostino–Pearson test confirmed that all data were normally distributed. All values are presented as means ± standard error of the mean (SEM). *p*‐values less than 0.05 were considered statistically significant.

## 3 RESULTS

### 3.1 ICS significantly reduced mechanical allodynia induced by peripheral nerve injury

**FIGURE 1:**
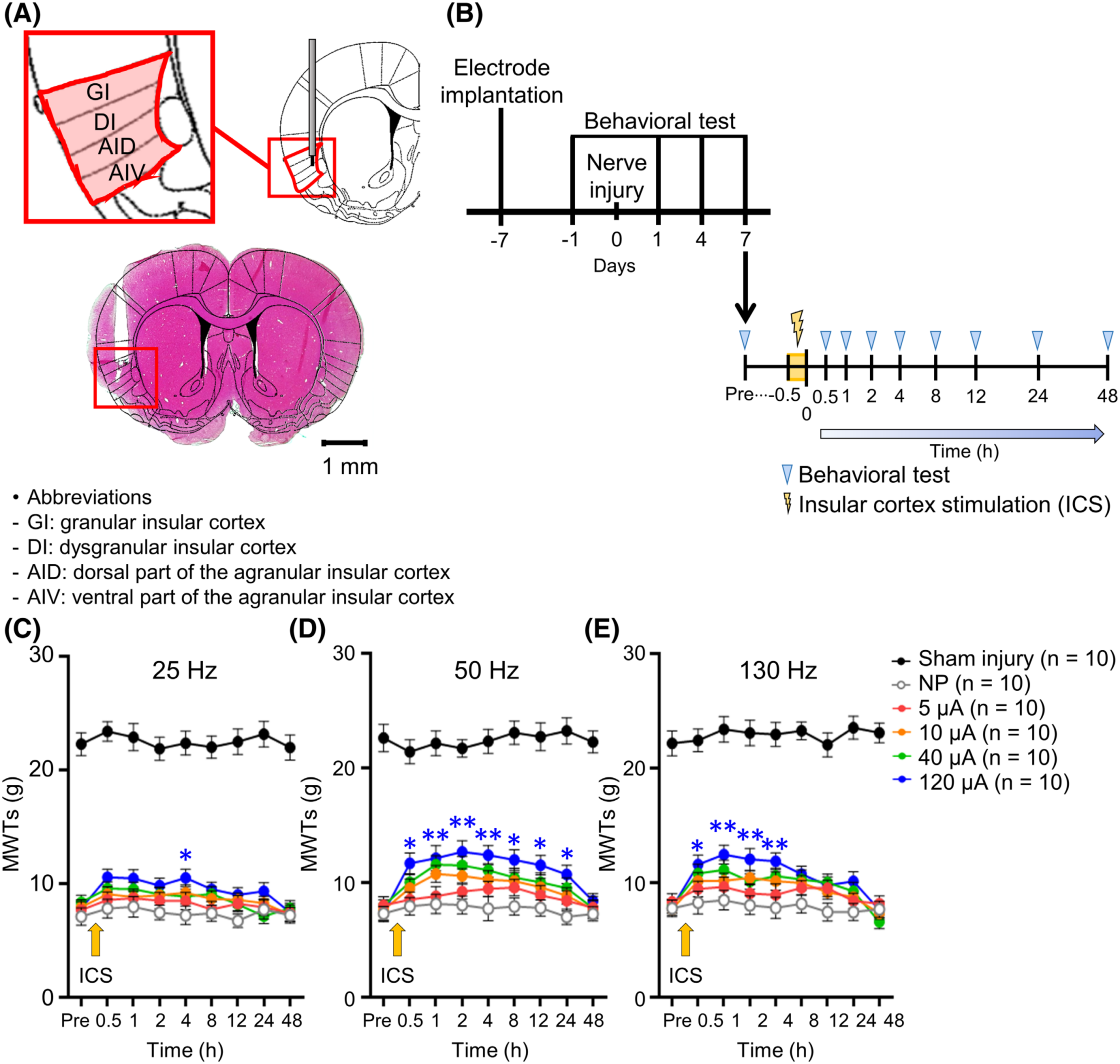
Single trial of insular cortex (IC) stimulation (ICS) increased mechanical withdrawal thresholds (MWTs). (A) Histological identification of the rostral anterior IC with rat atlas.^60^ Subdivisions of the IC are included in the red square box. The electrode was unilaterally implanted into the target site. (B) Schematic overview of the experimental procedure to observe the changes in MWTs on POD 7 depending on time points after applying single trial of ICS or sham stimulation. (C–E) Alteration of the MWTs after applying 25 (C), 50 (D), and 130 Hz (E) of a single trial of ICS on POD 7 was observed. (C) At 25 Hz, the attenuation effect of ICS on NP was revealed (for time: *F*_8,432_ = 3.154, *p* = 0.0018; for groups: *F*_5,54_ = 313.0, *p* < 0.0001; for time × group interaction: *F*_40,432_ = 0.4574, *p* = 0.9984, two‐way repeated‐measures ANOVA followed by Bonferroni test). Single trial of ICS at 25 Hz significantly increased MWTs only in 120 μA compared with NP group (*p* = 0.0407, Bonferroni's post hoc test). (D) At 50 Hz, a single trial of ICS was applied depending on several intensities of amplitudes, demonstrating a significant attenuation effect of ICS on NP (for time: *F*_8,432_ = 6.997, *p* < 0.0001; for groups: *F*_5,54_ = 179.8, *p* < 0.0001; for time × group interaction: *F*_40,432_ = 1.075, *p* = 0.3532, two‐way repeated‐measures ANOVA followed by Bonferroni test). The MWTs after single trial of ICS at 50 Hz–120 μA were significantly increased from 0.5 to 24 h (*p* < 0.01, Bonferroni's post hoc test). (E) After a single trial of ICS at 130 Hz, the MWTs significantly improved (for time: *F*_8,432_ = 7.945, *p* < 0.0001; for groups: *F*_5,54_ = 236.7, *p* < 0.0001; for time × group interaction: *F*_40,432_ = 1.061, *p* = 0.3744, two‐way repeated‐measures ANOVA followed by Bonferroni test). The MWTs were significantly increased in 130 Hz–120 μA group compared with NP group and maintained from 0.5 to 4 h (*p* < 0.01, Bonferroni's post hoc test). Data are presented as means ± SEM. *p < 0.05, **p < 0.01 vs. NP group, as determined using two‐way ANOVA followed by Bonferroni's post hoc multiple comparison test.

Figure 1A shows the actual position of the electrode that was inserted into the IC of the rats. A single trial of ICS with different parameters was applied to neuropathic rats on POD 7 (Figure 1B). To investigate the most effective parameters of ICS, the attenuation effects of ICS were shown depending on 25, 50, and 130 Hz (Figure 1C–E, respectively). As a result of a single trial of ICS at 25 Hz, the 120 μA group showed significantly increased MWTs at 4 h compared with the NP group; however, it did not last longer (Figure 1C, *p* < 0.05). The MWTs in the 5, 10, and 40 μA groups at 25 Hz did not significantly increase at any time point compared with those in the NP group. At 50 Hz, the MWTs in the 120 μA group were significantly improved and maintained from 0.5 to 24 h compared with those in the NP group (Figure 1D, *p* < 0.05). However, the 5, 10, and 40 μA groups at 50 Hz did not show significant attenuation effects of ICS at any time point compared with the NP group (*p* > 0.05). After a single trial of ICS at 130 Hz, the MWTs in the 120 μA group significantly improved from 0.5 to 4 h compared with those in the NP group (Figure 1E, *p* < 0.05). The MWTs in the 5, 10, and 40 μA groups at 130 Hz were not statistically significant compared with those in the NP group at all time points (*p* > 0.05). These results showed that ICS at 50 Hz–120 μA induced the most effective alleviation effect of NP, and the analgesic effects were maintained for 24 h in a single trial of ICS.

**FIGURE 2:**
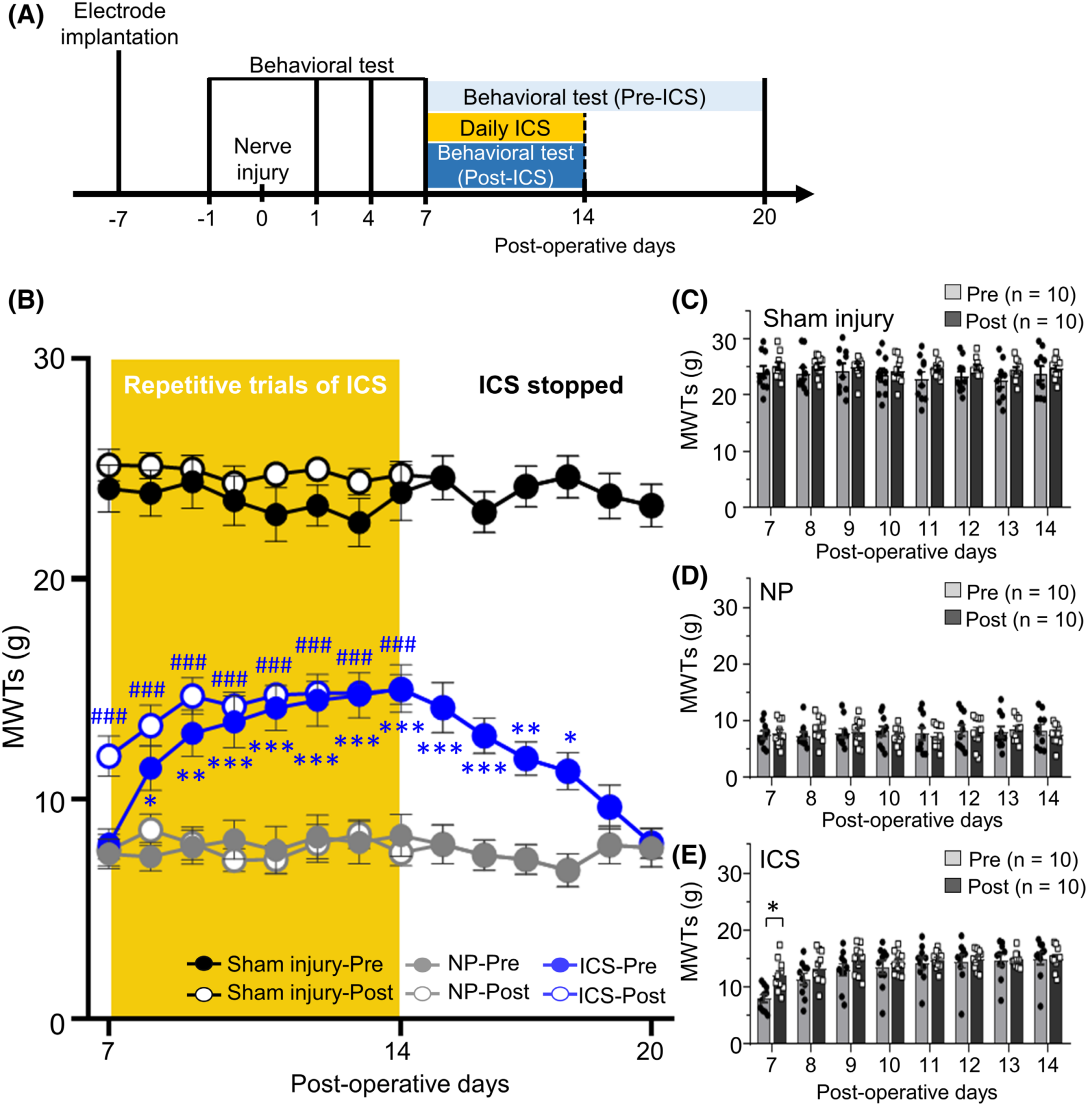
Repetitive trials of ICS reinforced the pain‐relieving effect. (A) Illustration of the experimental design on the repetitive trials of ICS. The MWTs from POD 7 to POD 14 were assessed before (Pre‐ICS) and after (Post‐ICS) repetitive trials of daily ICS or sham stimulation in sham injury, NP, and ICS groups. Repetitive trials of ICS or sham stimulation stopped from POD 15 to POD 20, with only behavior test being performed during this period. (B) The MWTs were measured before (Pre) applying daily ICS (repetitive trials of ICS, yellow background area) or sham stimulation and significantly increased from POD 8 to 14 compared with that in NP group. After (Post) repetitive ICS or sham stimulation, the MWTs were immediately measured and significantly increased from POD 7 to 14 (for postoperative days: *F*_13,351_ = 2.953, *p* = 0.0004; for groups: *F*_2,27_ = 248.2, *p* < 0.0001; for postoperative days × groups interaction: *F*_26,351_ = 2.932, *p* < 0.0001, two‐way repeated‐measures ANOVA followed by Bonferroni test). The most substantial attenuation effects were observed on POD 14 compared with those in the NP group (*p* < 0.0001, Bonferroni's post hoc test). Even after the ICS was stopped, the analgesic effects were maintained from POD 15 to POD 18 compared with those in the NP group (*p* < 0.001, Bonferroni's post hoc test). Data are presented as means ± SEM. *p < 0.05, **p < 0.01, ***p < 0.001 vs. NP‐Pre, ^#^ *p* < 0.05, ^##^ *p* < 0.01, ^###^ *p* < 0.001 vs. NP‐Post as determined using a two‐way ANOVA followed by Bonferroni's post hoc multiple comparison test. (C–E) The MWTs were compared before (Pre) and after (Post) daily ICS or sham stimulation in sham injury (C), NP (D), and ICS (E) groups. Additionally, the MWTs significantly changed immediately after daily ICS from POD 7 to POD 14 (for postoperative days: *F*_13,351_ = 4.603, *p* < 0.0001; for groups: *F*_2,27_ = 794.1, *p* < 0.0001; for postoperative days × groups interaction: *F*_26,351_ = 2.674, *p* < 0.0001, two‐way repeated‐measures ANOVA followed by Bonferroni test). (C) There were no significant differences in MWTs in sham injury (sham surgery + sham stimulation) group between before (Pre) and after (Post) sham stimulation (*p* > 0.05, *n* = 10, Bonferroni's post hoc test). (D) No significant differences in NP (neuropathic surgery + sham stimulation) group between before (Pre) and after (Post) sham stimulation (*p* > 0.05, *n* = 10, Bonferroni's post hoc test). (E) On POD 7, the MWTs after applying ICS were significantly increased compared with before (Pre) ICS (*p* < 0.05, *n* = 10, Bonferroni's post hoc test). Data are presented as means ± SEM. * *p* < 0.05 vs. Pre, as determined using two‐way ANOVA followed by Bonferroni's post hoc multiple comparison test.

The most effective parameters of ICS were selected at 50 Hz–120 μA. And then, the MWTs were measured before (Pre) and after (Post) repetitive trials of ICS or sham stimulation (Figure 2A). In ICS group, the MWTs were measured before applying repetitive daily ICS (ICS‐Pre) and significantly increased compared with the NP group from POD 8 to 14 (Figure 2B, *p* < 0.05). After daily ICS (ICS‐Post) during the period of ICS session, the MWTs were also significantly increased from POD 7 to 14 (*p* < 0.001). The most substantial attenuation effects were observed on POD 14. Even after ICS was stopped, the analgesic effects were maintained from POD 15 to POD 18 (*p* < 0.05). The MWTs were compared before (Pre) and after (Post) daily ICS or sham stimulation in sham injury, NP, and ICS groups (Figure 2C–E). In the sham injury and NP groups, there were no significant changes before (Pre) and immediately after (Post) sham stimulation from POD 7 to POD 14 (Figure 2C,D, *p* > 0.05). On POD 7 in the ICS group, the MWTs immediately after ICS (Post) were significantly increased compared with those before ICS (Pre) (*p* < 0.05), but there were no significant differences from POD 8 to 14 (Figure 2E, *p* > 0.05). These results indicated that prolonged attenuation effect of NP was observed when repetitive trials of the ICS were applied.

### 3.2 Changes in the expression level of pERK, neurons, astrocytes, and microglia in the IC after ICS

**FIGURE 3:**
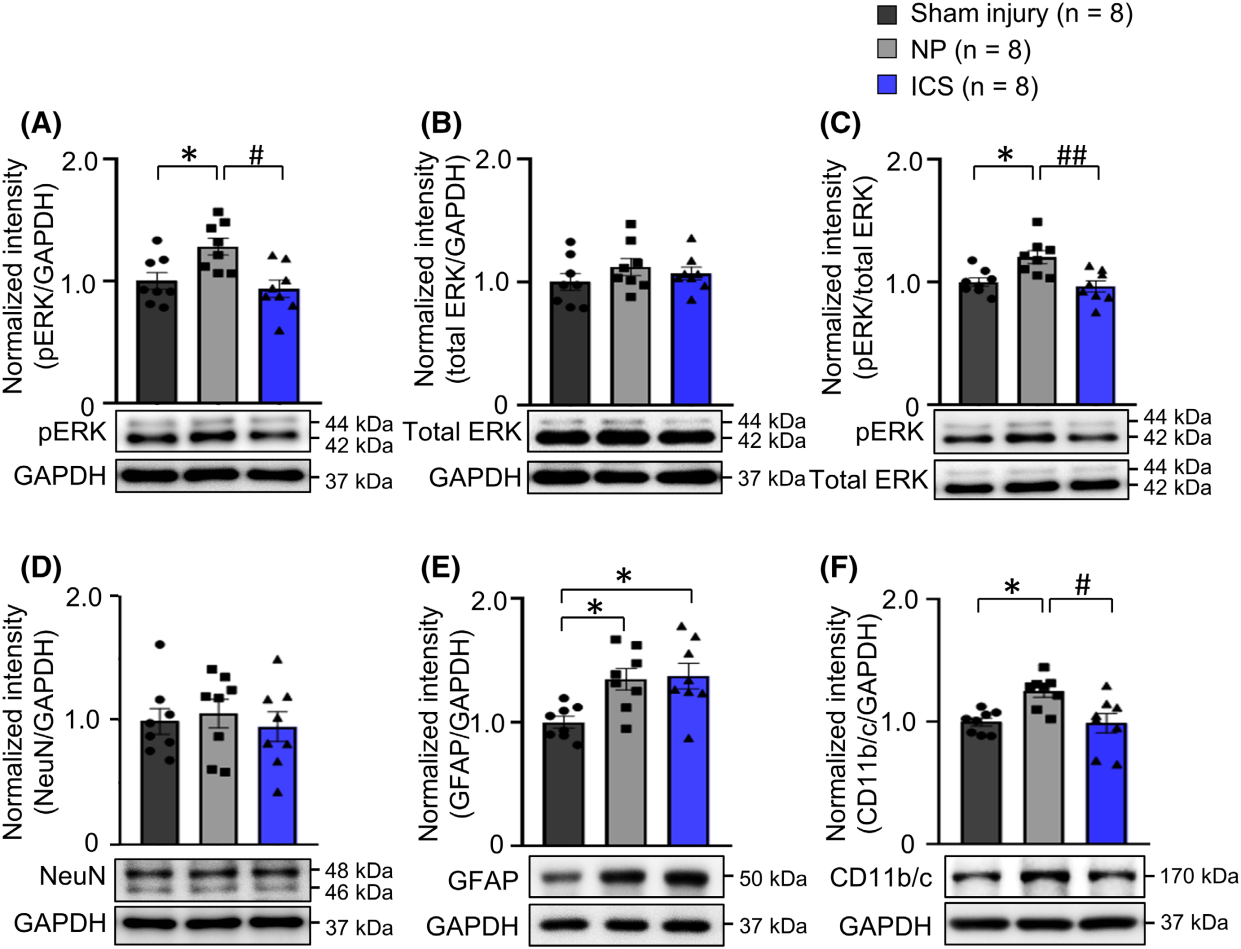
Molecular changes in pERK, neurons, astrocytes, and microglia after repetitive trials of ICS. (A) The expression level of pERK normalized to GAPDH showed significant changes (*F*_2,15_ = 6.730, *p* = 0.0082, one‐way ANOVA followed by Bonferroni test). The expression level of pERK in the NP group was significantly increased compared with that of the sham injury group (*p* < 0.05, *n* = 8). However, the expression level of pERK was significantly decreased in the ICS group compared with that in the NP group (*p* < 0.05, *n* = 8). (B) The expression level of total ERK was not changed between groups. (C) Significant changes in the expression level of pERK normalized to total ERK were observed (*F*_2,15_ = 6.791, *p* = 0.0079, one‐way ANOVA followed by Bonferroni test). The expression level of pERK normalized to total ERK in the NP group was increased compared with that in the sham injury group (*p* = 0.0471, *n* = 8, Bonferroni's post hoc test). However, it was decreased in the ICS group compared with that in the NP group (*p* = 0.0094, *n* = 8, Bonferroni's post hoc test). (D) The expression level of NeuN was not significantly changed in all groups (*F*_2,15_ = 0.8865, *p* = 0.4326, *n* = 8, one‐way ANOVA followed by Bonferroni test). (E) Significant differences between groups were observed in the expression levels of GFAP (*F*_2,15_ = 5.715, *p* = 0.0143, one‐way ANOVA followed by Bonferroni test). The expression levels of GFAP in the NP and ICS groups were significantly increased compared with those in the sham injury group (*p* = 0.0246, *n* = 8, Bonferroni's post hoc test), while no differences were observed between the NP and ICS groups (*p* = 0.9673, *n* = 8, Bonferroni's post hoc test). (F) Remarkable changes in the expression levels of CD11b/c were observed (*F*_2,15_ = 4.852, *p* = 0.0237, one‐way ANOVA followed by Bonferroni test). The expression level of CD11b/c in the NP group was increased compared with that in the sham injury group (*p* = 0.0477, *n* = 8, Bonferroni's post hoc test), while it was decreased in the ICS group compared with that in the NP group (*p* = 0.0361, *n* = 8, Bonferroni's post hoc test). Data are presented as means ± SEM. *p < 0.05 vs. sham injury group, ^#^ *p* < 0.05, ^##^ *p* < 0.01 vs. NP group as determined by using one‐way ANOVA followed by Bonferroni's post hoc multiple comparison test.

In order to assess the changes in the expression level of a marker for central sensitization in the IC, the expression level of pERK was validated following the repetitive trials of the ICS. The expression level of pERK normalized to GAPDH was significantly increased in the NP group than in the sham injury group (Figure 3A, *p* < 0.05). However, it was decreased in the ICS group compared with that in the NP group (*p* < 0.05). There were no significant changes in the expression levels of total ERK normalized to GAPDH among the groups (Figure 3B). The expression level of pERK normalized to total ERK in the NP group was significantly increased than that in the sham injury group (Figure 3C, *p* < 0.05). However, it was decreased in the ICS group compared with that in the NP group (*p* < 0.01).

To investigate the influence of ICS, the expression levels of neurons or glial cells were observed. There were no significant differences in the expression level of NeuN, a marker for neurons, between all groups (Figure 3D, *p* > 0.05). The expression level of glial fibrillary acidic protein (GFAP), a marker for astrocytes, was upregulated in the NP and ICS groups compared with that in the sham injury group (Figure 3E, *p* < 0.05). However, there were no significant differences between the NP and ICS groups (*p* > 0.05). In addition, the expression level of cluster of differentiation molecule 11b and c (CD11b/c), a marker for microglia, in the NP group was more increased than that in the sham injury group (Figure 3F, *p* < 0.05). However, it was decreased in the ICS group compared with that in the NP group (*p* < 0.05). These results revealed that ICS suppressed the upregulation of expression level of pERK and CD11b/c in the IC of neuropathic rats. However, the expression of astrocytes was increased after neuropathic surgery regardless of ICS. All the original images of unedited blots were provided in the Supplementary File S1 (Figure S1).

### 3.3 pERK expression was colocalized with neurons in the IC

**FIGURE 4:**
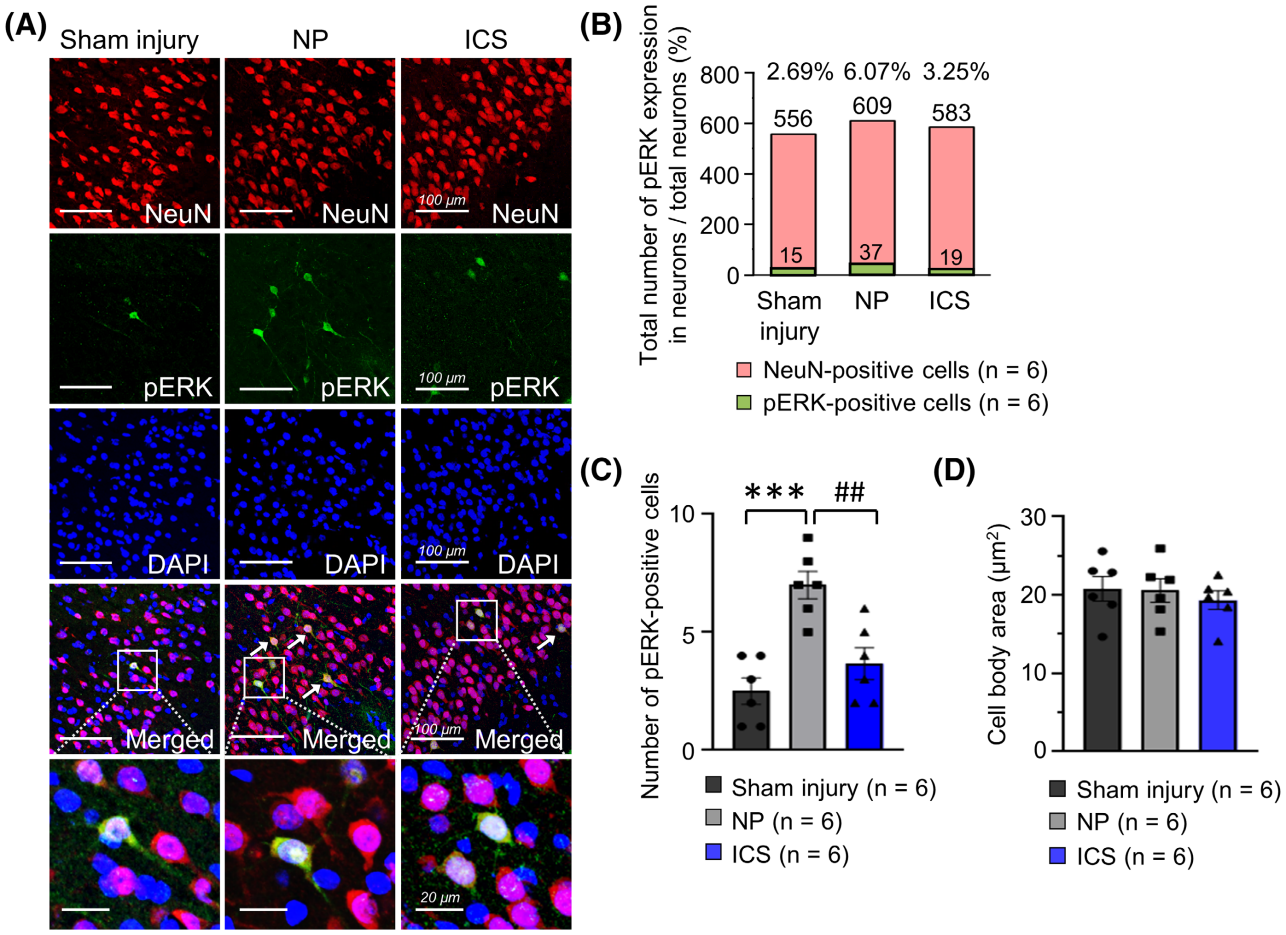
Colocalized expression of pERK and neurons was observed in the IC. (A) Representative images of immunofluorescence labeling of pERK and NeuN‐positive cells in sham injury, NP, and ICS groups. DAPI staining was performed to assess the cellular nuclei. Areas marked with a white box at the fourth row were magnified and placed at the bottom of merged images. Scale bar = 100 μm, 20 μm. (B) pERK‐ and NeuN‐positive cells were significantly correlated with each other (*χ* ^2^ = 9.956, *p* = 0.0069, chi‐squared test). The total number of pERK expressed in neurons showed above the green bar, and the total number of neurons located upper the red bar. These cells were counted manually in the IC of individual rats included in each group, and summed. (C) The number of pERK‐positive cells in the images showed significant changes (*F*_2,15_ = 14.95, *p* = 0.0003, one‐way ANOVA followed by Bonferroni test). The number of pERK‐positive cells in the NP group was significantly increased compared with that in the sham injury group (*p* = 0.0003, *n* = 6, Bonferroni's post hoc test). In ICS group, the number of pERK‐positive cells was substantially decreased (*p* = 0.0038, *n* = 6, Bonferroni's post hoc test). These cells were counted manually in the IC of individual rats included in each group, and averaged. Data are presented as means ± SEM and were analyzed using one‐way ANOVA followed by Bonferroni's post hoc test. (D) The size of the cell body area with NeuN‐positive cells was not changed in all groups (*p* > 0.05, *n* = 6). Data are presented as means ± SEM and were analyzed using one‐way ANOVA followed by Bonferroni's post hoc test. ***p < 0.001 vs. sham injury group, ^##^ *p* < 0.01 vs. NP group as determined by using one‐way ANOVA followed by Bonferroni's post hoc multiple comparison test.

Immunohistochemical staining was performed to determine the cell types that expressed pERK in the IC. The merged confocal images of sham injury, NP, and ICS groups showed colocalization of pERK‐positive cells and NeuN‐positive cells in the IC (Figure 4A). pERK‐ and NeuN‐positive cells were counted and calculated as percentage of pERK‐positive cells among neurons. Therefore, pERK‐positive cells among neurons showed that pERK‐ and NeuN‐positive cells were significantly correlated with each other (Figure 4B, *p* < 0.01). The number of pERK‐positive cells was significantly increased in NP group (Figure 4C, *p* < 0.001). However, it was significantly decreased after ICS (*p* < 0.01). Changes in the size of the cell body area of NeuN‐positive cells were also investigated to determine whether ICS affected the size of NeuN‐positive cells or not. The size of the cell body area of NeuN‐positive cells did not substantially change in any of the groups (Figure 4D, *p* > 0.05). Therefore, these results indicated that most pERK‐positive cells were colocalized with NeuN‐positive cells, irrespective of groups.

**FIGURE 5:**
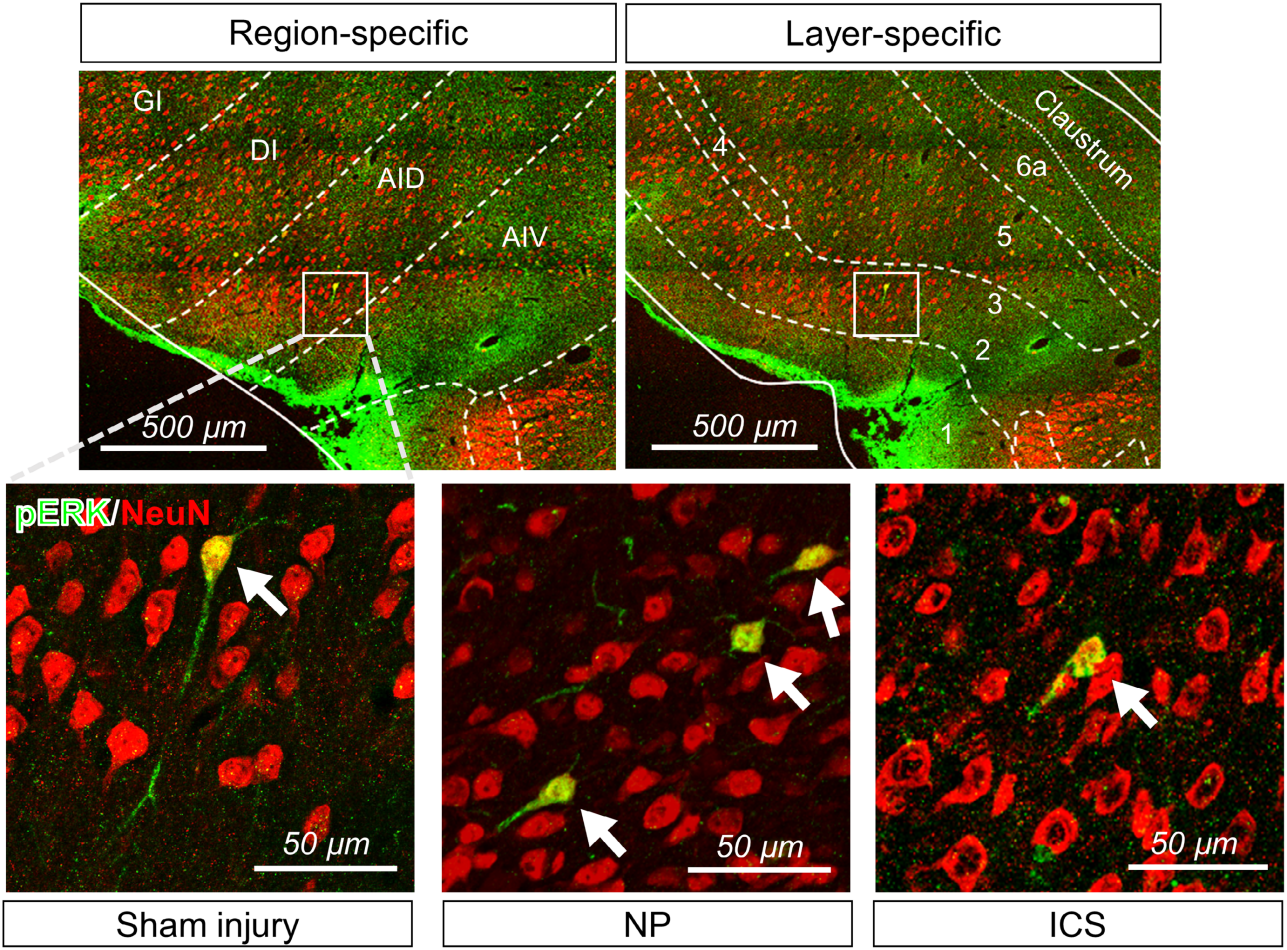
Region‐ or layer‐specific distribution of overlapped pERK‐positive cells and NeuN‐positive cells in the IC. Colocalized expression of pERK‐ and NeuN‐positive cells was mainly observed within the ventral or dorsal part of the agranular IC (top left) and located in layers 2–3 (top right) of the IC in the sham injury (bottom left), NP (bottom middle), and ICS groups (bottom right). Scale bar = 500 μm, 50 μm.

Furthermore, the colocalization of pERK‐positive cells with NeuN‐positive cells was observed in a region‐ and layer‐specific distribution in the IC (Figure 5). Colocalization of pERK‐ and NeuN‐positive cells was mainly observed within the ventral or dorsal part of the agranular IC (Figure 5, upper left) and also located in layers 2–3 of the IC (Figure 5, upper right). Regardless of the groups, the representative images showed the colocalization of pERK‐ and NeuN‐positive cells expressed within the ventral or dorsal part of the agranular IC and layers 2–3 of the IC (Figure 5, bottom).

### 3.4 pERK was not colocalized with astrocytes and microglia after ICS

**FIGURE 6:**
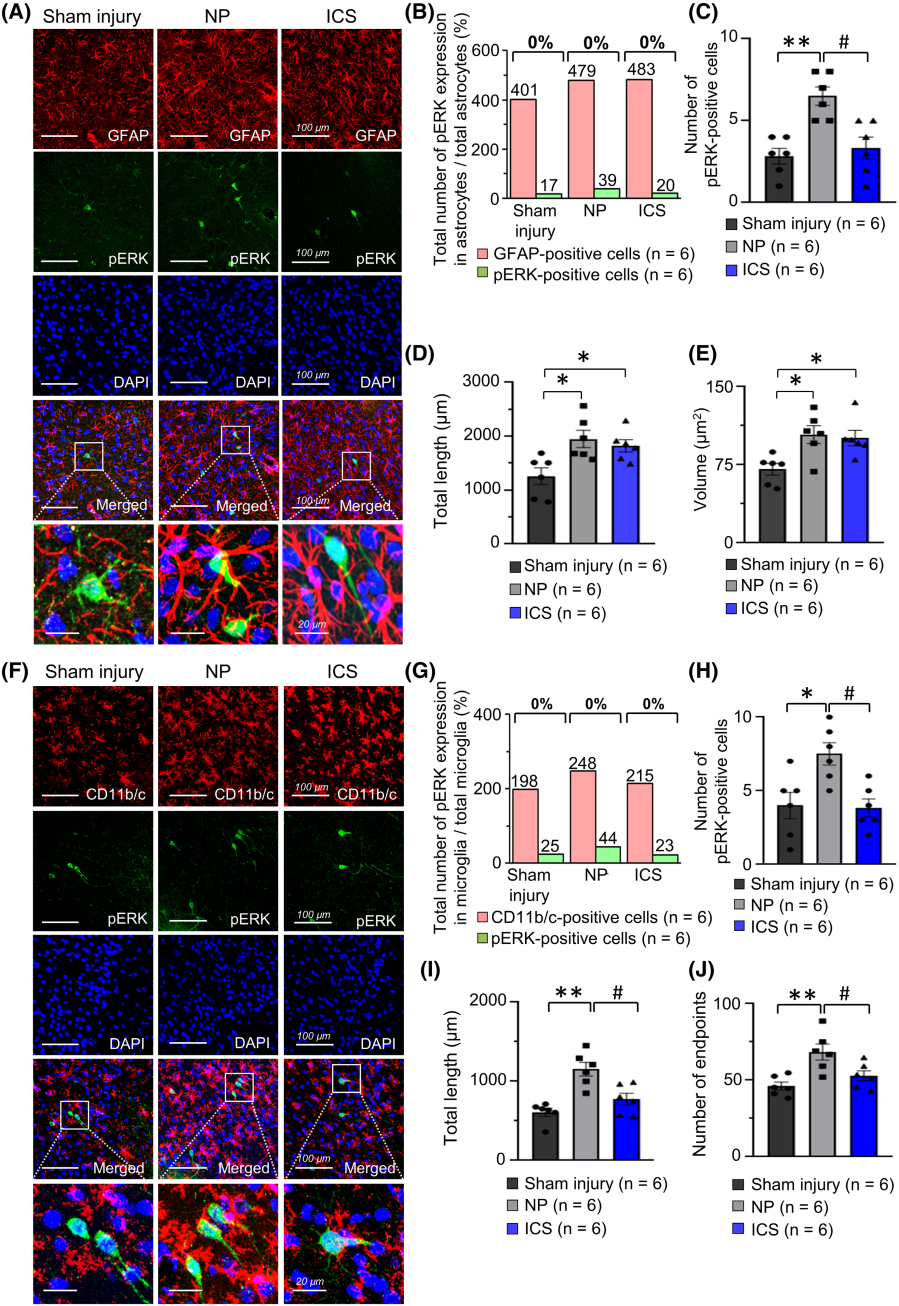
Immunofluorescence labeling of pERK and glial cells in the IC. (A) Representative images of immunofluorescence labeling of pERK and GFAP‐positive cells in sham injury, NP, and ICS groups. DAPI staining was performed to assess the cellular nuclei and the starting point for the analysis was set at the center of the DAPI‐stained nuclei. Area marked with a white box was magnified and placed at the bottom of merged image. Scale bar = 100 μm, 20 μm. (B) The expression of pERK‐positive cells was not colocalized with GFAP‐positive cells. These cells were not correlated each other (*χ* ^2^ = 0.9382, *p* = 0.6256, chi‐squared test). The total number of pERK expressed in astrocytes showed above the green bar, and the total number of astrocytes located upper the red bar. These cells were counted manually in the IC of individual rats included in each group, and summed. (C) The number of pERK‐positive cells in the NP group was significantly increased compared with that in the sham injury group (*p* < 0.05, *n* = 6). In ICS group, the number of pERK‐positive cells was significantly decreased compared with that in the NP group (*p* < 0.05, *n* = 6). These cells were counted manually in the IC of individual rats included in each group, and averaged. (D) The total length of GFAP‐positive cells showed significant changes between groups (*F*_2,15_ = 6.274, *p* = 0.0105, one‐way ANOVA followed by Bonferroni test). NP and ICS groups showed increases in the total length of the astrocytes in the IC compared with sham injury group (*p* < 0.05, *n* = 6, Bonferroni's post hoc test). There were no significant changes in total length of the astrocytes between NP and ICS groups. (E) The volume of GFAP‐positive cells confirmed significant differences between the groups (*F*_2,15_ = 6.445, *p* = 0.0095, one‐way ANOVA followed by Bonferroni test). The NP and ICS groups showed increases in the volume of the astrocytes in the IC compared with that in the sham injury group (*p* < 0.05, *n* = 6, Bonferroni's post hoc test). There were no significant changes in the volume of astrocytes between NP and ICS groups. Total length and volume were quantified by Sholl analysis protocol.^31^ (F) Representative images of immunofluorescence labeling of pERK and CD11b/c‐positive cells in sham injury, NP, and ICS groups. DAPI staining was performed to assess the cellular nuclei and the starting point for the analysis was set at the center of the DAPI‐stained nuclei. Area marked with a white box was magnified and placed at the bottom of merged image. Scale bar = 100 μm, 20 μm. (G) The expression of pERK‐positive cells was not colocalized with CD11b/c‐positive cells. These cells were not correlated each other (*χ* ^2^ = 0.0366, *p* = 0.9818, chi‐squared test). The total number of pERK expressed in microglia showed above the green bar, and the total number of microglia located upper the red bar. These cells were counted manually in the IC of individual rats included in each group, and summed. (H) The number of pERK‐positive cells in the images showed significant changes (*F*_2,15_ = 5.504, *p* = 0.0161, one‐way ANOVA followed by Bonferroni test). The number of pERK‐positive cells in the NP group was substantially increased compared with that in the sham injury group (*p* < 0.05, *n* = 6). In the ICS group, the number of pERK‐positive cells was significantly decreased (*p* < 0.05, *n* = 6). These cells were counted manually in the IC of individual rats included in each group, and averaged. (I) The total length of CD11b/c‐positive cells was significantly changed (*F*_2,15_ = 14.66, *p* = 0.0003, one‐way ANOVA followed by Bonferroni test). The NP group showed significant increase in the total length of microglia‐positive cells compared with the sham injury group (*p* = 0.0071, *n* = 6, Bonferroni's post hoc test). The ICS group showed decreases in total length compared with the NP group (*p* = 0.006, *n* = 6, Bonferroni's post hoc test). (J) Significant changes in the number of endpoints were observed (*F*_2,15_ = 8.694, *p* = 0.0031, one‐way ANOVA followed by Bonferroni test). The number of endpoints was significantly increased in the NP group compared with that in the sham injury group (*p* = 0.003, *n* = 6, Bonferroni's post hoc test). Decreases in the number of endpoints were observed in the ICS group compared with those in the NP group (*p* = 0.0376, *n* = 6, Bonferroni's post hoc test). *p < 0.05, **p < 0.01 vs. sham injury group, ^#^ *p* < 0.05, ^##^ *p* < 0.01 vs. NP group as determined by using a one‐way ANOVA followed by Bonferroni's post hoc multiple comparison test. Data are presented as means ± SEM.

To investigate the colocalization of pERK and glial cells, immunohistochemistry was conducted. The merged images of cells in the sham injury, NP, and ICS groups showed that pERK‐ and GFAP‐positive cells were not colocalized (Figure 6A). Therefore, pERK‐ and GFAP‐positive cells were not overlapped and correlated with each other (Figure 6B, *p* > 0.05). This result showed that pERK‐positive cells were not immunoreactive for GFAP‐positive cells in the IC. The number of pERK‐positive cells was significantly increased in the NP group than that in the sham injury group (Figure 6C, *p* < 0.01). However, after repetitive ICS, the number of pERK‐positive cells was substantially decreased than that in the NP group (*p* < 0.05). The total length of GFAP‐positive cells in the NP (*p* < 0.05) and ICS groups (*p* < 0.05) significantly increased compared with that in the sham injury group (Figure 6D). There were no significant differences in the total length of GFAP‐positive cells between the NP and ICS groups (*p* > 0.05). In addition, the volume of GFAP‐positive cells in the NP (*p* < 0.05) and ICS groups (*p* < 0.05) was significantly increased than that in the sham injury group (Figure 6E, *p* < 0.05). However, there were no significant differences in volume between the NP and ICS groups (*p* > 0.05).

The merged confocal images of cells in the sham injury, NP, and ICS groups showed that pERK‐ and CD11b/c‐positive cells were not overlapped (Figure 6F). Therefore, pERK‐positive cells among microglia confirmed that these cells were not correlated with each other (Figure 6G, *p* > 0.05). The number of pERK‐positive cells was significantly increased in the NP group than that in the sham injury group (*p* < 0.05), and it was substantially decreased after repetitive ICS than that in the NP group (Figure 6H, *p* < 0.05). The total length of CD11b/c‐positive cells in NP group was increased compared with the sham injury group (*p* < 0.01); however, the total length of CD11b/c‐positive cells was reduced in the ICS group than that in the NP group (Figure 6I, *p* < 0.05). The number of endpoints in CD11b/c‐positive cells was increased in the NP group compared with sham injury group (*p* < 0.01), but it was decreased after ICS (Figure 6J, *p* < 0.05).

## 4 DISCUSSION

### 4.1 ICS at 50 Hz–120 μA induced the most effective alleviation effect on NP

Some studies reported that electrophysiological recordings in neuropathic rats showed markedly increased firing rates in the IC, and it was alleviated when the NP was attenuated.^33^, ^34^ Similarly, voltage‐sensitive dye imaging studies showed that higher peak amplitudes and larger activation areas in the IC were observed in neuropathic rats than normal status, and it was ameliorated after modulation of the mammalian target of rapamycin complex in the IC.^19^, ^35^ Also, ICS induces antinociception by functionally modulating opioid and cannabinoid systems.^11^ Therefore, we could infer that ICS at 50 Hz–120 μA might have reduced excessive IC activation caused by NP and induced pain modulation.

Although both the 25 Hz–120 μA and 130 Hz–120 μA groups showed significant pain‐relieving effects, the improved MWTs in the 25 Hz–120 μA and 130 Hz–120 μA did not last longer than those in the 50 Hz–120 μA group. Accumulating evidence suggests that brain stimulation at lower frequencies, such as 25 Hz used in this study, did not show a significant attenuation effect in chronic pain because it might not be sufficient to trigger changes in neuronal firing of cortical regions.^36^, ^37^ In the case of higher frequency (>100 Hz), the stimulation of cortical regions resulted in excessively increased neuronal activity during stimulation, and a regional inactivation effect was temporarily maintained after stimulation was turned off.^13^, ^34^, ^38^ Therefore, these results suggest that lower frequency could not be induce the sufficient changes in neuronal activity of the IC for pain modulation, and a high frequency might produce a temporarily regional inactivation of IC in neuropathic rats but not lasted longer. Furthermore, no substantial attenuation effects were observed at intensities lower than 120 μA. A previous study^39^ reported that sufficient modification of neuronal firing is dependent on amplitude intensity. These results indicated that the higher intensity of amplitudes, rather than lower intensity, in the IC changed neuronal firing, leading to pain‐relieving effects in NP.

The present study showed that repetitive daily ICS from POD 7 to POD 14 induced significantly improved and prolonged attenuation effects for 4 days longer even after the termination of ICS. According to previous studies, NP leads to changes in molecular to synaptic levels owing to increased signal transduction which results in the formation of synaptic plasticity.^2^, ^22^ Besides, previous studies have suggested a possibility that brain stimulation affects the synaptic plasticity modulation by controlling neurotransmitter transporters or synaptic transmission.^40^, ^41^ In line with the accumulating evidence, our results suggest that the repetitive ICS might modulate the synaptic plasticity.

### 4.2 Repetitive ICS modulated the expression of pERK in the neurons of the IC, but not glial cells

We investigated whether ICS induced alterations in pERK expression levels in the IC of neuropathic rats. The expression level of pERK in NP group was significantly increased compared with that in the sham injury group. After repetitive ICS, the expression levels of pERK decreased compared with those in the NP group. It has been known that pERK expression depends on stimulus intensity and is induced by high‐intensity noxious stimuli under normal conditions.^24^, ^42^ However, in pathological pain conditions, pERK expression is significantly increased in response to innocuous and low‐intensity stimuli due to spontaneous and abnormal signals.^24^, ^43^ When NP was alleviated, the expression levels of pERK were reduced.^35^, ^44^ These results suggest that pERK expression is increased under NP conditions through activated cascades or pathways and decreased by the attenuation of spontaneous and abnormal signals after repetitive ICS, resulting in pain‐relieving effects in the present study.

In this study, the expression of pERK was colocalized with neurons in the IC and it is consistent with previous study.^45^ According to previous study, the nociceptive‐specific neurons were located in layers 2–3 of the IC,^45^ and other studies reported that these neurons were classified as pyramidal neurons.^26^, ^46^ Therefore, these cells did not respond to innocuous stimuli in physiological conditions, and excessively activated in NP status.^26^, ^45^, ^46^ These neurons might potentially express pERK due to the astrocytes located near neurons.^26^, ^46^ These astrocytes participate in signal transduction between neurons under NP conditions and release inflammatory mediators to express pERK within neurons.^45^, ^47^ These results suggest that, for pain modulation, pERK expression might be modulated in neurons in layers 2–3 of the IC in neuropathic rats due to influence of the inflammatory mediators from astrocytes.

Interestingly, the present study showed that the expression of pERK was not colocalized with astrocytes or microglia in the IC of neuropathic rats. Previous studies reported that colocalization of pERK and astrocytes leads to maintenance of NP^26^, ^27^, ^28^ and contributes to reactive astrogliosis by production of pro‐inflammatory cytokines and inflammatory mediators for sustained late‐phase NP.^48^, ^49^ Conversely, GFAP‐positive cells devoid of pERK suggest that sustained activation of the ERK pathway may not contribute to some aspects of reactive astrogliosis.^48^, ^49^ Therefore, these results indicated that pERK expression in the IC was not overlapped with astrocytes and it might not contribute to reactive astrogliosis in response to NP. pERK within microglia was associated with microglial proliferation, adoption of an effector morphology, and development of cold or mechanical pain‐related hypersensitivity.^50^ Overlap of pERK expression with microglia was mostly observed in the acute phase of pain development because microglia are involved in proinflammatory mediators and release of cytokines in response to injury.^28^, ^51^ These results suggested that pERK expression is not colocalized with microglia on POD 14 and may not contribute to microglia activation such as proliferation or morphology.

### 4.3 Repetitive ICS did not affect to astrocytic activation under neuropathic rats

In this study, the expression level of astrocytes was increased in the NP and ICS groups compared with that in the sham injury group, but there were no significant differences in GFAP expression between the NP and ICS groups. Astrocytes actively respond to NP conditions because they surround the synapses of neurons and are involved in the transmission of pain signals.^52^, ^53^ Increased astrocytic activity after brain stimulation may be involved in glutamate release for the activation of calcium signaling or the pre‐/postsynaptic adenosine 1 receptor modulation for inhibition of neuronal communication.^6^, ^54^, ^55^ These results indicated that astrocytes are in an active state in the IC of neuropathic rats, and repetitive ICS also might lead to increasing astrocytic activation via a different pathway such as activation of calcium signaling or inhibition of neuronal communication for pain‐relieving effects.

### 4.4 Repetitive ICS might normalize the microglia in NP conditions

The present study showed that the expression levels of microglia were increased in NP conditions and decreased after repetitive ICS. Nerve injuries induced the morphological changes in microglia of the higher subcortical regions at least more than 1 week after injury, accompanied by drastic increased expression of pro‐inflammatory genes.^56^, ^57^ Therefore, the present study indicated that the expression level of microglia in the IC might be affected by the increased expression of pro‐inflammatory genes on POD 14. Also, previous studies have reported that normalized microglia density, soma size, and activation were observed in the area around the electrode implantation after brain stimulation.^58^, ^59^ These changes were affected by the suppression of pro‐inflammatory cytokine levels and promotion of anti‐inflammatory cytokine expression.^58^, ^59^ These results indicated that ICS could modulate the changes in morphology or in the expression level of microglia through the interference of inflammatory responses under NP conditions.

In conclusion, the present study showed that ICS effectively alleviated NP. The expression level of pERK was reduced by ICS, and pERK was colocalized with neurons in layers 2–3 of the IC. This preclinical study may enhance the potential use of ICS and may serve as a translational platform to reveal therapeutic mechanisms of ICS in NP.

## AUTHOR CONTRIBUTIONS

Bae Hwan Lee and Myeounghoon Cha conceptualized and supervised this study. Kyeongmin Kim performed behavioral test, western blot, immunohistochemistry, and data analyses and also wrote the manuscript. Guanghai Nan performed hematoxylin–eosin staining for histology. Leejeong Kim performed image acquisition via confocal microscope. Minjee Kwon and Kyung Hee Lee performed neuropathic surgery and electrode implantation. All authors revised the manuscript.

## FUNDING INFORMATION

This study was supported by a National Research Foundation of Korea (NRF) grant funded by the Korean Government (MSIT) NRF 2016R1D1A3B2008194 (K.H.L.), NRF 2019R1I1A1A01059697 (M. C.), and NRF 2020R1A2C3008481 (B.H.L.).

## CONFLICT OF INTEREST STATEMENT

The authors declare that they have no known competing financial interests or personal relationships that could have appeared to influence the work reported in this paper.

## Supporting information


Figure S1.

## Data Availability

The data that support the findings of this study are available from the corresponding author upon reasonable request.
