# Supplementary material for: Insular cortex stimulation alleviates neuropathic pain via ERK phosphorylation in neurons
Source: CNS Neurosci Ther. 2023 Feb 20;29(6):1636–48. doi: 10.1111/cns.14126 (PMC10173725; doi:10.1111/cns.14126)
Supplement: Supplementary file 1 — Figure S1. [file CNS-29-1636-s001.pdf]

Supplemental Files

Insular cortex stimulation alleviates neuropathic pain via ERK phosphorylation in neurons

Kyeongmin Kim [1], Guanghai Nan [1,2], Leejeong Kim [1,2], Minjee Kwon [3], Kyung Hee Lee [4], Myeounghoon Cha [1*], Bae Hwan Lee [1,2*]

[1] Department of Physiology, Yonsei University College of Medicine, Seoul 03722, Republic of Korea

[2] Department of Medical Science, Brain Korea 21 Project, Yonsei University College of Medicine, Seoul 03722, Republic of Korea

[3] Department of Nursing, Kyungil University, Gyeongsan 38428, Republic of Korea

[4] Department of Dental Hygiene, Division of Health Science, Dongseo University, Busan 47011, Republic of Korea;

* Corresponding authors

Myeounghoon Cha, PhD and Bae Hwan Lee, PhD

Department of Physiology, Yonsei Unive/rsity College of Medicine,
50-1, Yonsei-ro, Seodaemun-gu, Seoul 03722, Korea

Tel.: +82 2-2228-1711

E-mail: mhcha@yuhs.ac and bhlee@yuhs.ac

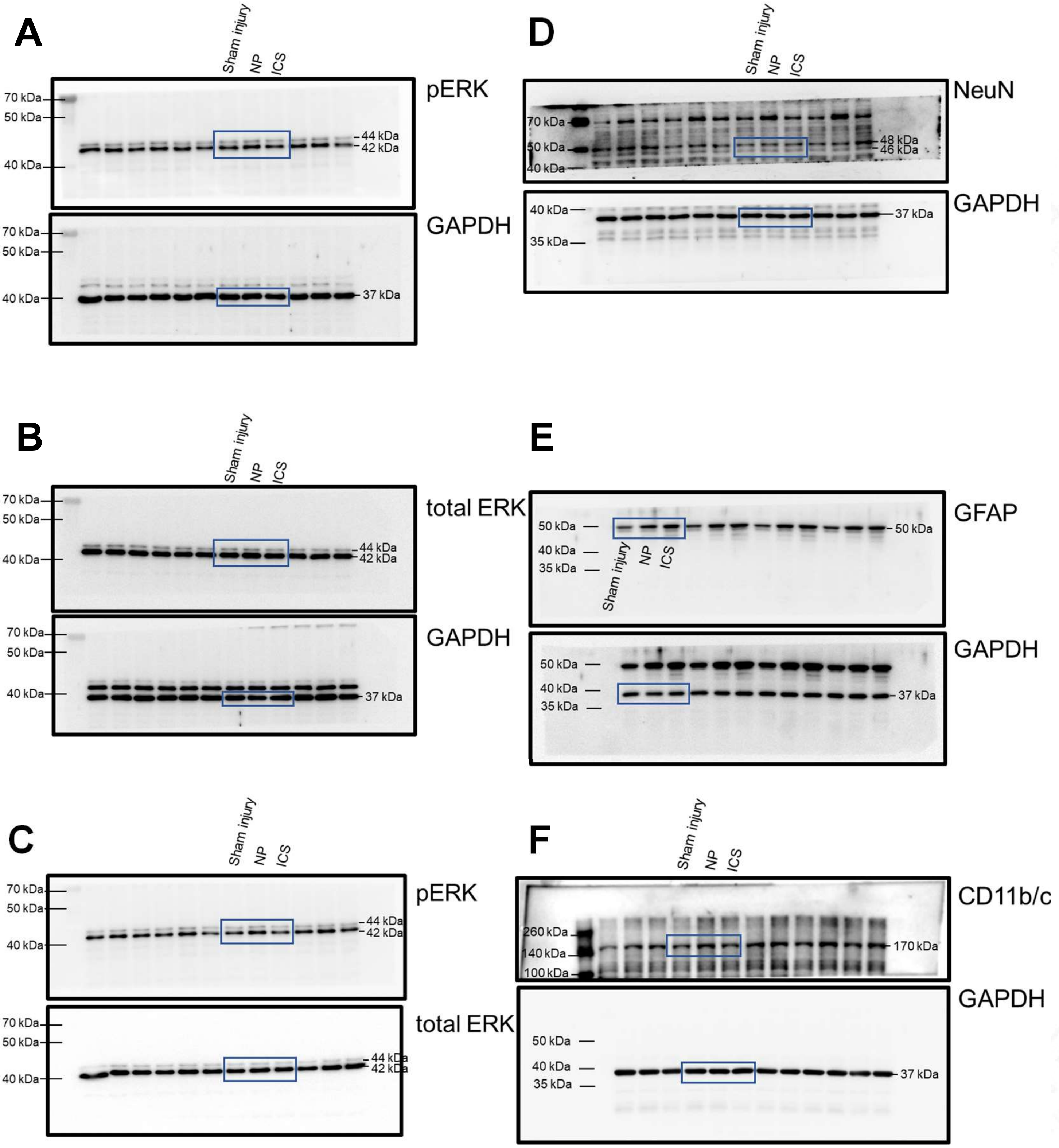

Supplementary File S1. Each unedited blots of western blotting shown in Figure 3. Punctured IC tissues were subjected to western blotting with pERK, total ERK, pERK/total ERK, NeuN, GFAP or CD11b/c antibodies. Anti-GAPDH antibody was used as a loading control. The blue boxes in original images were used in Figure 3.
